# Protective effect of *Ganoderma lucidum*-fermented crop extracts against hydrogen peroxide- or β-amyloid-induced damage in human neuronal SH-SY5Y cells

**DOI:** 10.1186/s12906-024-04409-1

**Published:** 2024-04-05

**Authors:** Chung-Hsiung Huang, Yu-Ting Liao, Chien-Li Chen, Guo-Jane Tsai

**Affiliations:** 1 Department of Food Science, National Taiwan Ocean University, 2 Pei-Ning Road, Keelung, 202 Taiwan ROC; 2 Center for Marine Bioscience and Biotechnology, National Taiwan Ocean University, Keelung, Taiwan

**Keywords:** Acetylcholinesterase, Amyloid plaques, Anti-oxidation, *Ganoderma lucidum*, Solid-state fermentation

## Abstract

**Background:**

Alzheimer's disease (AD) is a neurodegenerative disorder characterized by the accumulation of stacked β-amyloid peptides in the brain and associated with the generation of oxidative stress. So far, there is no cure for AD or a way to stop its progression. Although the neuroprotective effects of *Ganoderma lucidum* aqueous extract and *G. lucidum*-derived triterpenoids and polysaccharides have been reported, the influence of *G. lucidum*-fermented crops on AD still lacks clarity.

**Methods:**

This study aimed to investigate the protective effect of *G. lucidum*-fermented crop extracts against hydrogen peroxide- or β-amyloid peptide (Aβ_25-35_)-induced damage in human neuroblastoma SH-SY5Y cells.

**Results:**

Various extracts of *G. lucidum*-fermented crops, including extract A: 10% ethanol extraction using microwave, extract B: 70˚C water extraction, and extract C: 100˚C water extraction followed by ethanol precipitation, were prepared and analyzed. Extract B had the highest triterpenoid content. Extract C had the highest total glucan content, while extract A had the highest gamma-aminobutyric acid (GABA) content. The median inhibitory concentration (IC_50_, mg/g) for DPPH and ABTS scavenging activity of the fermented crop extracts was significantly lower than that of the unfermented extract. Pretreatment with these extracts significantly increased the cell viability of SH-SY5Y cells damaged by H_2_O_2_ or Aβ_25-35_, possibly by reducing cellular reactive oxygen species (ROS) and malondialdehyde (MDA) levels and increasing superoxide dismutase (SOD), glutathione peroxidase (GPx) and catalase (CAT) activities. Moreover, extract B markedly alleviated the activity of acetylcholinesterase (AChE), which is crucial in the pathogenesis of AD.

**Conclusion:**

These results clearly confirmed the effects of *G. lucidum*-fermented crop extracts on preventing against H_2_O_2_- or Aβ_25-35_-induced neuronal cell death and inhibiting AChE activity, revealing their potential in management of AD.

**Supplementary Information:**

The online version contains supplementary material available at 10.1186/s12906-024-04409-1.

## Background

Alzheimer's disease (AD) is a progressive and chronic neurodegenerative disease [1]. Recent studies indicate that AD accounts for a significant portion of dementia cases worldwide, with the prevalence projected to increase dramatically by 2050 [2]. The disease is characterized by neurofibrillary tangles and senile plaques composed of aggregated β-amyloid peptide and metal-ions, with oxidative stress and free radicals playing a pivotal role in its development [3]. Oxidative damage caused by the interaction of redox active metal ions with β-amyloid can lead to neuronal cell death and neurodegeneration [3].

Currently, treatment for AD mainly revolves around cognition-enhancing medications, but these drugs can only temporarily relieve symptoms and can cause adverse effects to patients [4]. Many therapeutic strategies have been studied for AD, one of which is the cholinergic hypothesis. According to this hypothesis, enhancing cholinergic neurotransmission may improve cognitive function in AD patients [5]. Inhibitors of acetylcholinesterase (AChE), responsible for the breakdown of acetylcholine, have emerged as a potential treatment [6]. However, existing AChE inhibitors have their limitations in terms of adverse effects and bioavailability [4].

Natural products, such as those found in *G. lucidum*, a Basidiomycetes fungus with a long history of medicinal use in East Asia, have attracted attention for their potential as selective AChE inhibitors [7]. *G. lucidum* is known for its diverse bioactive compounds, including polysaccharides, triterpenes, phenolics, and flavonoids, which contribute to its health-promoting effects and antioxidant properties [8–10]. Although research on the beneficial properties of *Ganoderma* has been extensive, only a few studies have investigated the neuroprotective potential of *Ganoderma* aqueous extracts and *Ganoderma*-derived triterpenoids and polysaccharides [11–16]. Given the critical role of neurodegenerative diseases in AD, this study focused on investigating whether *G. lucidum* fermented crop extract can alleviate oxidative stress and Aβ_25-35_-induced neurotoxicity, thereby providing a nutraceutical that can prevent neurodegenerative diseases.

Previous studies have revealed the neuroprotective potential of wild *G. lucidum* fruiting bodies [12–16]. Due to the scarcity of wild *G. lucidum* resources, artificial solid media are used to cultivate *G. lucidum* fruiting bodies. When *G. lucidum* grows in solid culture media, it first produces mycelium, and only after light induction does it grow fruiting bodies. This process takes 3–5 months. In contrast, cultivation of *G. lucidum* in liquid medium with shaking takes only 2–3 weeks to harvest mycelium and extracellular polysaccharides [17]. In this study, we used an edible crop mixture as a solid medium to culture *G. lucidum* mycelium. Various extracts of solid-state fermented crops were prepared and their bioactive substance contents were determined. The antioxidant activity of these extracts in scavenging free radicals and protecting human neuronal SH-SY5Y cells damaged by H_2_O_2_ or Aβ_25-35_ were evaluated. The modulatory effects of these extracts on intracellular antioxidant enzymes and AChE activities were elucidated to provide the potential mechanisms of action. Furthermore, these effects of fermented crop extracts were compared with those of unfermented crop extracts and GABA, which is considered a potential candidate for AD treatment [18]

## Materials and methods

### Culture, chemicals and reagents

*G. lucidum* BCRC 36123 was purchased from the Bioresources Collection and Research Center (Hsinchu, Taiwan). SH-SY5Y cell line (ATCC CRL-2266) was kindly provided by Professor Wen-Mei Fu in Pharmacological Institute, College of Medicine, National Taiwan University. Unless specified otherwise, all chemicals and reagents used in the experiments were obtained from Sigma-Aldrich Chemical Co. (St. Louis, MO, USA). Reagent for cell culture was purchased from Thermo Fisher Scientific Inc. (Waltham, MA, USA). Hydrogen peroxide was purchased form Honeywell International, Inc. (Charlotte, NC, USA). GABA standard was purchased from ChromaDex Inc. (Longmont, CA, USA). Catalase (CAT), glutathione peroxidase (GPx), superoxide dismutase (SOD) and thiobarbituric acid reactive substances (TBARS) assay kits were purchased from Cayman chemical (Ann Arbor, MI, USA). Acetylcholinesterase assay kit was purchased from Abcam (Trumpington, Cambridge, UK).

### Solid-state fermentation of crops by *G. lucidum* and extracts preparation

Forty gram of crop mixture (containing embryo rice and wheat germ in a weight ratio of 6:4) and equal amount of water were added into a jar (diameter 6.5 cm × height 12.5 cm), which was then sterilized at 121 °C for 15 min to prepare the crop medium. At the same time, *G. lucidum* BCRC 36123 was cultured on Gano medium agar plate at 30 °C for 7 days, and then, 5 mL sterile water was added to wash the spores of *G. lucidum* [19]. This spore suspension was inoculated into the crop medium in the jar to have initial spore density of 10^5^ spores/g. After culturing at 30 °C for 21 days, the fermentation product was freeze-dried, homogenized, and extracted by the following three methods. Since the fermented crop products may contain the bioactive components of triterpenes, GABA, and polysaccharides (glucans), we refer some specifically triterpenes (method A), GABA (method B) and glucans (method C) extracted report to try to extract specifically each ingredient to study which ingredient is responsible for neuroprotective activity. For method A, the fermented crop powder (15 g) was added with 10% ethanol (300 mL), heated by a microwave at 300 W for 10 min [20] and centrifuged (4600 g, 20 min). The collected supernatant was lyophilized and named extract A. For method B, the fermented crop powder (15 g) was added with distilled water (300 mL), mixed with vortex for 10 min and then heated in water bath at 70˚C for 30 min [21]. After centrifugation (4600 g, 20 min), the supernatants were mixed with four volume of 95% ethanol and kept at 4˚C overnight to precipitate polysaccharides. After centrifugation (4600 g, 20 min), the collected supernatants were lyophilized and named extract B. For method C to prepare extract C, it is similar to method B, except that the heating conditions are 100 °C for 5 h, and the precipitate is collected by centrifugation after ethanol treatment.

### Measurement of mycelium and proximate composition in *G. lucidum*-fermented crops

The amount of *G. lucidum* mycelium in the fermented crops was analyzed based on the method described by Han et al. by analyzing the ergosterol amount in samples [22]. The proximate compositions of the extracts, which encompassed crude ash, crude fat, and crude protein, were evaluated following the procedures outlined in the AOAC (1990). To determine the carbohydrate content, the values for crude ash, fat, and protein were subtracted from the total dry matter content [23].

### Determination of bioactive components in *G. lucidum*-fermented crop extracts

The contents of total glucan, α-and β-glucan in the extracts were determined by Mushroom and Yeast beta-glucan Assay (Megazyme Ltd., Wicklow, Ireland) according to the supplier’s instruction. To determine the content of triterpenes, the extracts (1 g) were mixed with 50 mL of 10% ethanol and heated by microwave at 300W for 10 min. The supernatant was obtained through centrifugation at 4600 g for 10 min. The quantification of total triterpenes content was carried out using a colorimetric reaction described by Lin et al. [20]. This reaction involves vanillin-perchloric acid and glacial acetic acid, and it allows for the determination of triterpenes present in the sample. Based on the method described by Chen et al*.* [24], the GABA contents in the extracts were analyzed. Briefly, 1 g extract powder was shaken with 0.1 mol/L HCl for 45 min and filtered through Whatman No. 4 filter paper. This filtrate was reacted with o-phthalaldehyde reagent (Sigma-Aldrich Chemical Co.) and then immediately injected into HPLC. The HPLC system consisted of a Shimadzu LC-10AT VP pump, a Rheodyne 7725i injector, a 20-μl sample loop, a Shimadzu RID-10A detector, and a Myghtysil RP-18 GP column (4.6 × 250 mm, 5 μm, Kanto Chemical Co., Inc., Tokyo, Japan). Mobile phases were A, methanol; B, 0.5% (v/v) acetic acid in 0.1 M sodium acetate. The gradient of A:B is 60:40, which lasts from 0 to 16 min, and then the phase B is slowly reduced to 0 at a flow rate of 1 mL/min. The optical density (O.D.) was read at 345 nm. HPLC profiles of GABA content are shown in Fig. S1. Total soluble phenolics in extracts were determined using Folin-Ciocalteu reagent method and using gallic acid as a standard phenolic compound [25]. The outcome data were expressed as mg gallic acid equivalents (GAE)/g of dry samples. Based on the method described by Ćilerdžić et al.[9], the flavonoid content in sample was analyzed using the reagent mixture of aluminum nitrate and potassium acetate. Quercetin was used as a standard and outcome data were expressed as mg quercetin equivalents (QE) /g of dry sample.

### Evaluation of free radical scavenging capacity of *G. lucidum*-fermented crop extracts

The 1,1-diphenyl-2-picrylhydrazyl (DPPH) scavenging activity of sample was measured as described previously with some modification [26]. 100 μL of extracts with different concentrations, dissolved in ethanol, was mixed with 100 μM of DPPH (2,2-diphenyl-1-picrylhydrazyl) ethanol solution (500 μL). The mixture was incubated in the dark for 30 min. Then the absorbance at 517 nm was measured. Ascorbic acid was used as a standard. The capability to scavenge the DPPH radical was calculated using the following equation:

$$DPPH\;scavenging\;activity\;(\%)\:=\:\lbrack(A_{control}-A_{sample})/A_{control}\rbrack\:\times\:100\%$$

The 2,2'-azino-bis(3-ethylbenzothiazoline-6-sulfonic acid) (ABTS) scavenging activity of sample was measured as described previously with some modification [27]. Briefly, the reagents of 20 μL of ABTS (740 μM), 20 μL of H_2_O_2_ (750 μM) and 20 μL of peroxidase (44 units/mL) were mixed and incubated at room temperature in the dark for 12 h. Then, 40 μL of various concentrations of extract solution was added and reacted for 10 min. The absorbance at 734 nm was measured and Trolox was used as the standard. The capability to scavenge the ABTS radical was calculated using the following equation:

$$ABTS\;scavenging\;activity\;(\%)\:=\:\lbrack(A_{control}-A_{sample})/A_{control}\rbrack\:\times\:100\%$$

### Preparation of Aβ_25-35_ plaque

As described by Kowall *et. al*., Aβ_25-35_ peptides dissolved in sterilized deionized water (1 mM) were incubated at 37 °C for 7 days [28]. To confirm the formation of plaque, 50 μL of cultured Aβ_25-35_ solution was added with 1 mL of Thioflavin-T (5 mM in 50 mM glycine–NaOH solution at pH 8.5) to label β-sheet structure. Next, the plaque fluorescence was observed using a fluorescence microscope (Olympus IX71) at a magnification of × 200. The excitation wavelength was 450 nm, and the emission wavelength was 482 nm. The sample of Aβ_25-35_ plaque was stored at -20 °C until the beginning of cell culture experiments.

### Cell culture experiments

Since undifferentiated SH-SY5Y cells were found to be more susceptible to Aβ_25-35_ than differentiated cells, we chose to use undifferentiated SH-SY5Y cells in this study [29]. It has been suggested that the Aβ_25-35_ peptide is the bioactive domain of Aβ_1-42_ [30]. This fragment is widely used by neuroscience researchers to establish AD models in vitro and in vivo [31, 32]. Furthermore, a previous study provided compelling evidence that Aβ_1-42_ and Aβ_25-35_ peptides induce neural damage in similar patterns, establishing Aβ_25-35_ as a convenient tool to study the mechanisms of neurotoxicity associated with AD [33]. Therefore, Aβ_25-35_ was used in this study. GABA is known to have neuroprotective activity [34], so it was used for comparison with the treatment group. To investigate the influence of various extracts per se on cell viability, SH-SY5Y cells (1 × 10^4^ cells/well) were cultured in 96-well plates with 200 µL of DMEM medium containing 10% fetal bovine serum, 1% streptomycin/penicillin and 1% streptomycin/penicillin and incubated for 24 h. Subsequently, 10 μL of different concentrations of extracts were added to the cells and further incubated for 24 h. To assess the preventive effect of extracts against cell damage induced by H_2_O_2_ or Aβ_25-35_, SH-SY5Y cells were cultured at a density of 1 × 10^4^ cells per well for 24 h. Then, various concentrations of extracts were added and incubated for 6 h, followed by addition of 10 μL H_2_O_2_ (150 μM) or Aβ_25-35_ plaque (10 μM) and incubated for another 24 h [35]. Finally, the cells were collected and washed with sterile phosphate-buffered saline (PBS) twice. The cell viability was measured using 3-(4,5-dimethylthiazol-2-yl)-2,5-diphenyl-tetrazolium bromide (MTT) assay [36]. Viability (%) = (O.D. value of treated cells/O.D. value of untreated cells) × 100%. On the other hand, the intracellular ROS and MDA production, and SOD, GPx, CAT, and AchE activities were determined as previously described [28, 35, 36]. Briefly, cultured SH-SY5Y cells were suspended in lysis buffer and centrifuged (15,000 g, 20 min) at 4 °C to collect the supernatant. The protein concentration was determined using Bio-Rad protein assay kit. After reacting with 20 μmol/L DCFH-DA for 30 min, the ROS level in the supernatant was measured using a fluorescence spectrometer [37]. Cellular SOD, GPx, CAT and AchE activities were determined spectrophotometrically using relevant commercial kits [38]. Cellular MDA production was determined using a commercial TBARS assay kit [39]. These assays were performed according to the manufacturer's instructions.

Meanwhile, SH-SY5Y cells that were sample-treated and then treated with H_2_O_2_- or Aβ_25-35_ as described above were washed twice with PBS and then stained with Hoechst 33,342 dye (10 μg/mL) for 10 min [40]. By observing with fluorescence microscopy (Olympus IX71), apoptotic cells with a nuclear pattern of condensed chromatin can be distinguished from the uniform pattern of normal cells.

### Statistical analysis

Statistical analysis of the data was performed using SPSS Version 12.0 (SPSS Inc., Chicago, IL, USA). To assess statistical differences between sample means, one-way analysis of variance (ANOVA) was employed, with the significance level set at *p* < 0.05. Post hoc multiple comparisons of means were conducted using Duncan’s multiple range test. All results are presented as mean ± standard deviation (SD).

## Results

### Composition analysis of *G. lucidum*-fermented crop powder and various extracts

After fermentation, the *G. lucidum* mycelium amount in the fermented crops was 214.12 ± 1.31 mg/g, as expressed as ergosterol amount in sample [22]. The crude protein, crude fat and ash contents in fermented crops increased significantly, while the carbohydrate content decreased significantly (Table 1). These results show that carbohydrates are used as carbon sources for *Ganoderma* growth. The higher protein, lipid and mineral contents of *G. lucidum* mycelium in fermented crops may lead to increased protein, lipid and ash contents in fermented crops [20].

**Table 1:**
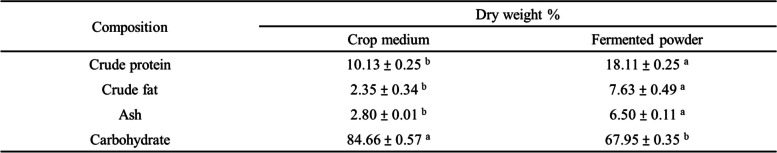
Composition (dry weight basis) of crop medium and *G. lucidum-*fermented powder

The data were presented as mean ± SD from three separate experiments. Different letters (a-b) within the same group indicate a significant difference (*p* < 0.05). The carbohydrate content was calculated as 100% minus the sum of crude protein, crude fat, and ash content

Originally, based on some reports, we used specific triterpene (Method A), GABA (Method B), and polysaccharide (Method C) extraction protocols were used to try to specifically extract each component to investigate which component is responsible for the neuroprotective activity. However, as shown in Table 2, none of the three extraction methods specifically extracted the main bioactive components. All 3 extracts contained glucans, triterpenes, phenolics, peptides, and flavonoids. Except for peptides, the contents of glucan, triterpenes, GABA, peptide, phenolics and flavonoids in the fermented powder were significantly increased, compared to those in unfermented crop medium (Table 2). Noticeably, Extract A contained the highest level of GABA, phenolics and flavonoids; Extract B contained the highest level of triterpenes; Extract C contained the highest level of total glucan, phenolics and flavonoids, but no GABA (Table 2).

**Table 2:** Bioactive substance content in crop medium, *G. lucidum*-fermented powder and its various extracts

Contents	Crop medium	Fermented powder	Extracts		
			A	B	C
Total glucan (g/100 g)	6.97 ± 0.65 ^d^	36.80 ± 0.90 ^b^	6.27 ± 0.06 ^d^	20.13 ± 0.36 ^c^	60.73 ± 0.98 ^a^
α-glucan (g/100 g)	3.42 ± 0.05 ^d^	17.13 ± 0.73 ^a^	4.39 ± 0.24 ^c^	4.11 ± 0.24 cd	15.54 ± 0.36 ^b^
β-glucan (g/100 g)	3.55 ± 0.70 ^d^	19.67 ± 1.59 ^b^	1.88 ± 0.22 ^e^	16.02 ± 0.30 ^c^	45.19 ± 1.19 ^a^
Triterpenes (mg/g)	1.43 ± 0.20 ^e^	10.80 ± 0.20 ^d^	16.39 ± 0.87 ^b^	20.30 ± 0.24 ^a^	12.25 ± 0.72 ^c^
GABA (mg/g)	0.62 ± 0.01 ^d^	1.34 ± 0.04 ^c^	1.85 ± 0.04 ^a^	1.40 ± 0.03 ^b^	ND
Peptide content (mg/g)	0.15 ± 0.01 ^b^	0.12 ± 0.03 ^b^	0.20 ± 0.03 ^a^	0.04 ± 0.02 ^c^	0.02 ± 0.01 ^c^
Phenolics (mg GAE/g)	6.97 ± 0.11 ^e^	13.52 ± 0.78 ^d^	28.40 ± 0.90 ^a^	26.60 ± 0.62 ^b^	23.53 ± 0.74 ^c^
Flavonoids (mg QE/g)	ND	0.86 ± 0.40 ^c^	3.08 ± 0.04 ^a^	2.04 ± 0.12 ^b^	1.68 ± 0.36 ^b^

The data were presented as mean ± SD from three separate experiments. Different letters (a-e) within the same group indicate a significant difference (*p* < 0.05)

Extract A, fermented powder extracted with 10% ethanol using microwave extraction. Extract B, fermented powder extracted with 70 °C water, followed by 95% ethanol precipitation and collect supernatant. Extract C, fermented powder with 100 °C water, followed by 95% ethanol precipitation and collect precipitate

*GAE* Gallic acid equivalent, *QE* Quercetin equivalent, *ND* Not detectable

### Free radical scavenging capacity and antioxidant activities of *G. lucidum*-fermented crop extracts

As free radical and oxidative stress plays an important role in the pathogenesis of AD, the free radical scavenging capacity and antioxidant activities of fermented crop extracts were investigated. The half inhibitory concentration (IC_50_) of DPPH and ABTS free radical scavenging activities of fermented crop powder were significantly lower than those of unfermented crop culture medium (Table 3). Furthermore, the IC_50_ values f DPPH and ABTS for extract A were significantly lower than those of fermented crop, with the lowest IC_50_ value of DPPH radical scavenging activity being observed in extract A (Table 3).

**Table 3:**
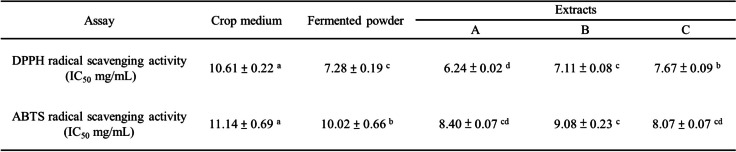
Antioxidant activity of in crop medium, *G. lucidum*-fermented powder and its various extracts

The data were presented as mean ± SD from three separate experiments. Different letters (a-d) within the same group indicate a significant difference (*p* < 0.05)

**Fig. 1:**
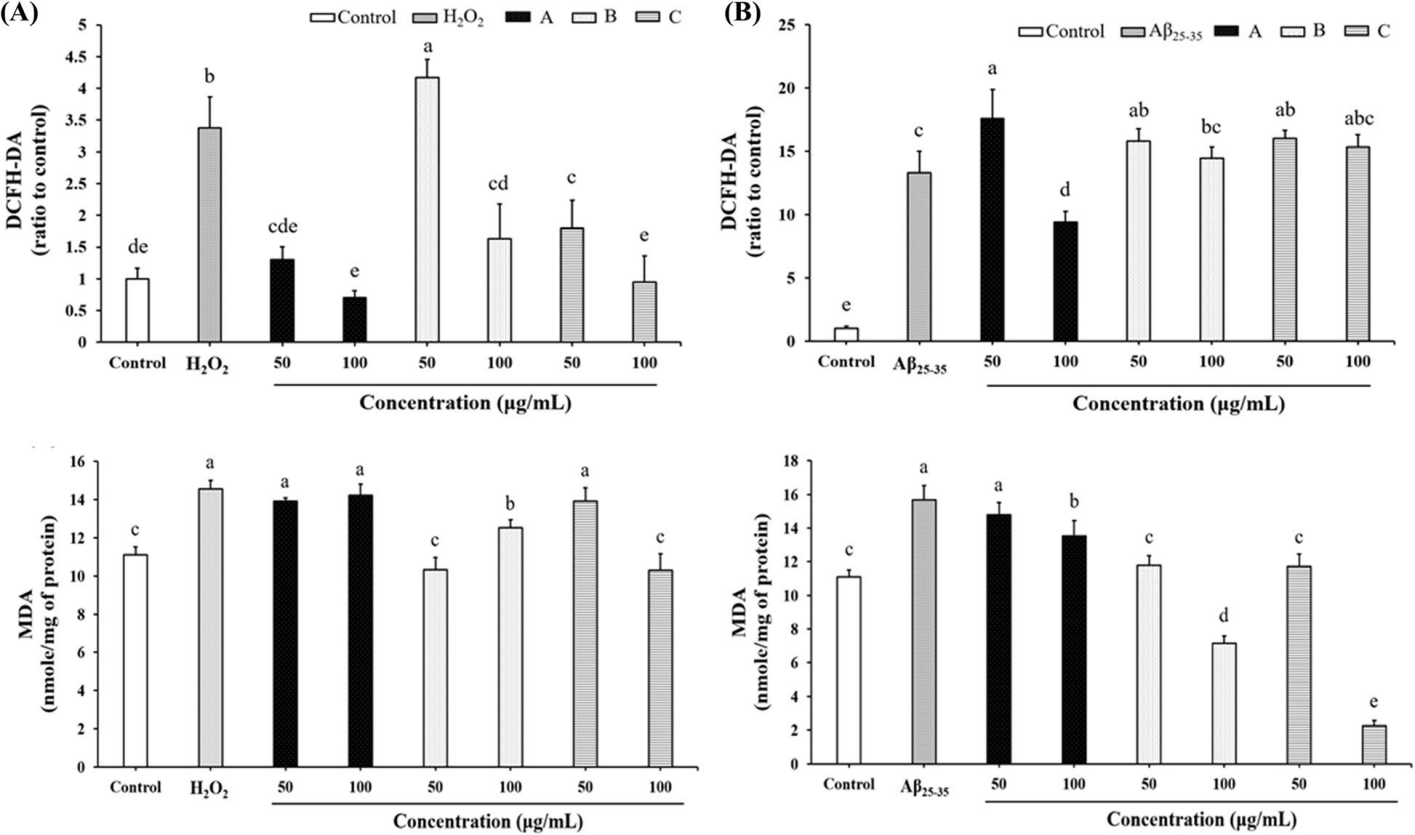
Influence of *G. lucidum*–fermented crop extracts on the levels of reactivity oxygen species (ROS) and malondialdehyde (MDA) in (**A**) H_2_O_2_- or (**B**) Aβ_25-35_-treated SH-SY5Y cells. Cells pretreated with various *G. lucidum*–fermented crop extracts for 6 h followed by either H_2_O_2_ (150 μM) or Aβ_25-35_ (10 μM) treatment for 24 h. The level of ROS and MDA were determined by DCFH-DA and TBARS assays, respectively, as described in Materials and methods. The data are represented as mean ± SD from three independent experiments. In the same figure, different letters (a-e) denote significant differences (*p* < 0.05) among the groups being compared

In the cell culture experiment, we first evaluated the effects of various concentrations of fermented crop extracts on cell viability using the MTT method. No significant reduction in cell viability was observed in SH-SY5Y cells treated with non-fermented crop extracts or fermented crop extracts at concentrations of 0–500 μg/mL, except for Extract C at a concentration of 500 μg/mL (Fig. S2). Therefore, extract concentrations of 0–100 μg/mL were chosen to evaluate their effects on intracellular ROS and MDA production in H_2_O_2_ or Aβ_25-35_-treated cells. On the other hand, the required concentration of H_2_O_2_ or Aβ_25-35_ for the following in vitro experiments was also explored through MTT assay. As shown in Fig. S3, cell viabilities at 150 and 200 μM of H_2_O_2_ treatment were 80.0% and 54.2%, respectively; while cell viabilities at 10 and 15 μM of Aβ_25-35_ treatment were 71.1% and 63.3%, respectively. Therefore, the concentration of H_2_O_2_ at 150 μM and that of Aβ_25-35_ at 10 μM were chosen for the induction of oxidative stress and cell damage. Pre-treatment with these three extracts could attenuate H_2_O_2_-induced intracellular ROS production (Fig. 1A). Pre-treatment of extract B and extract C even diminished the level of MDA (Fig. 1A). Pretreatment of extract A at 100 μg/mL significantly reduced ROS production in Aβ_25-35_-treated SH-SY5Y cells, whereas pretreatment of extract B and extract C had limited effect on preventing ROS production (Fig. 1B). Furthermore, pretreatment with the 3 tested extracts significantly reduced the amount of MDA in Aβ_25-35_-treated SH-SY5Y cells (Fig. 1B).

### Effects of *G. lucidum*-fermented crop extracts on ameliorating H_2_O_2_- or Aβ_25-35_-induced SH-SY5Y cell death

**Fig. 2:**
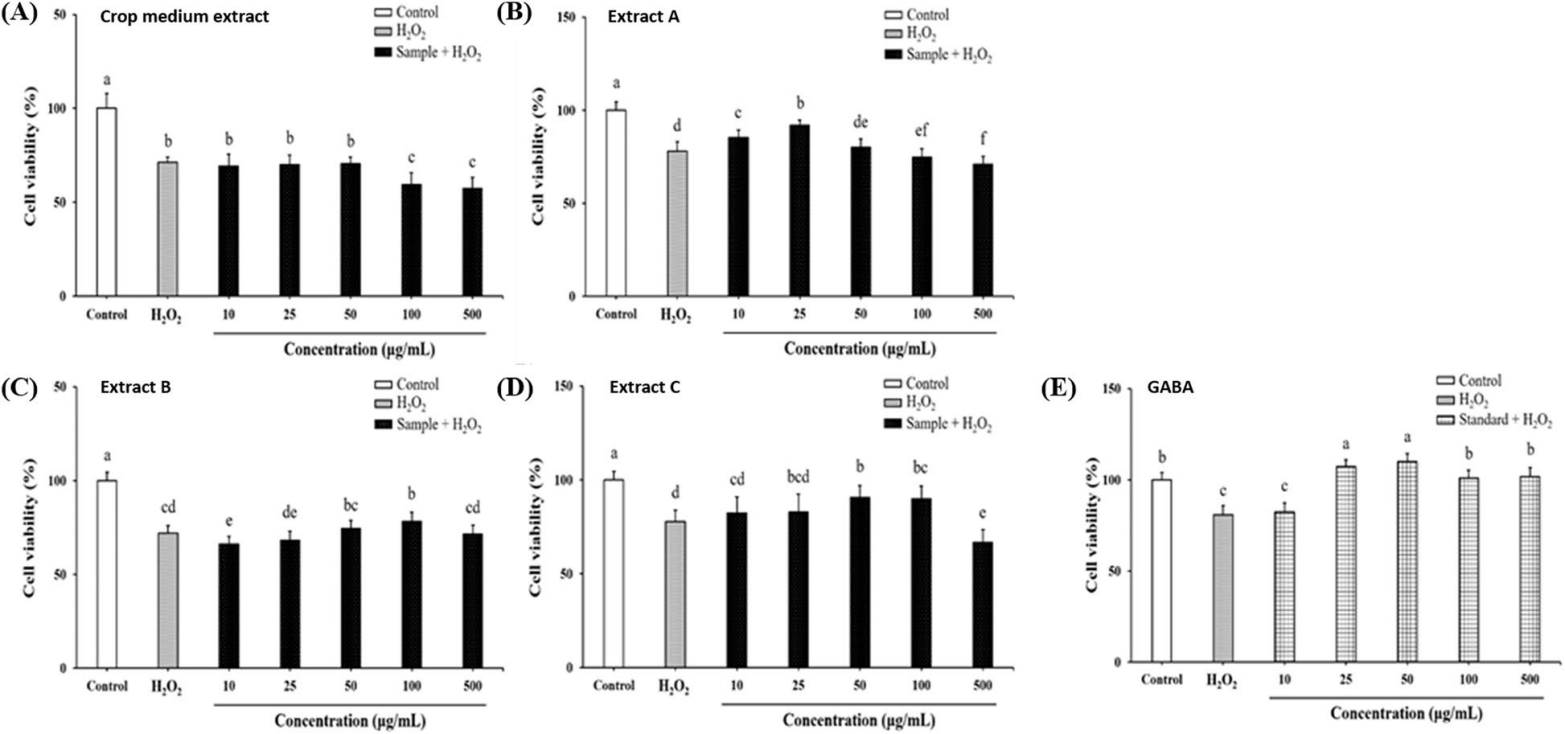
The protective effects of *G. lucidum*-fermented crop extracts against H_2_O_2_-induced cytotoxicity in SH-SY5Y cells were evaluated. The cells were pre-treated with various substances: (**A**) Crop medium extract, (**B**) *G. lucidum*-fermented crop extract A, (**C**) extract B, (**D**) extract C, and (**E**) GABA standard (at concentrations ranging from 10 to 500 μg/mL) for 6 h, followed by treatment with H_2_O_2_ (150 μM) for an additional 24 h. Cell viability was assessed using the MTT assay, as detailed in the Materials and methods section. The data were expressed as mean ± SD from three independent experiments. Different letters (a-e) displayed within the same figure indicate significant differences (*p* < 0.05) among the various treatment groups

**Fig. 3:**
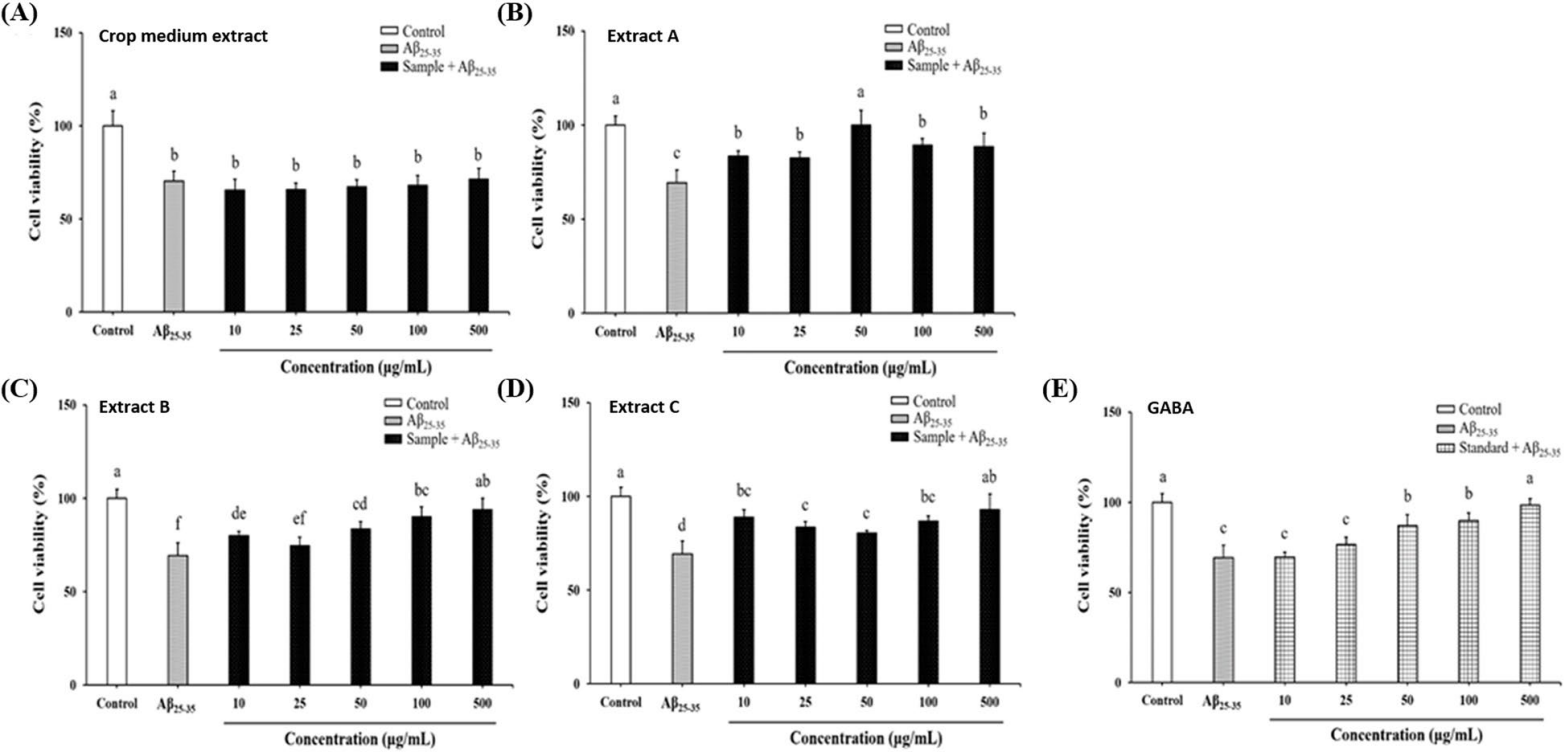
Effects of *G. lucidum*-fermented crop extracts on protecting against Aβ_25-35_-induced cytotoxicity in SH-SY5Y cells. The cells were pre-treated with different substances: (**A**) Crop medium extract, (**B**) *G. lucidum*-fermented crop extract A, (**C**) extract B, (**D**) extract C, and (**E**) GABA standard (at concentrations ranging from 10 to 500 μg/mL) for 6 h, followed by treatment with Aβ_25-35_ (10 μM) for an additional 24 h. Cell viability was assessed using the MTT assay, following the procedures outlined in the Materials and methods section. The data were expressed as mean ± SD from three independent experiments. Different letters (a-f) shown within the same figure indicate significant differences (*p* < 0.05) among the various treatment groups

**Fig. 4:**
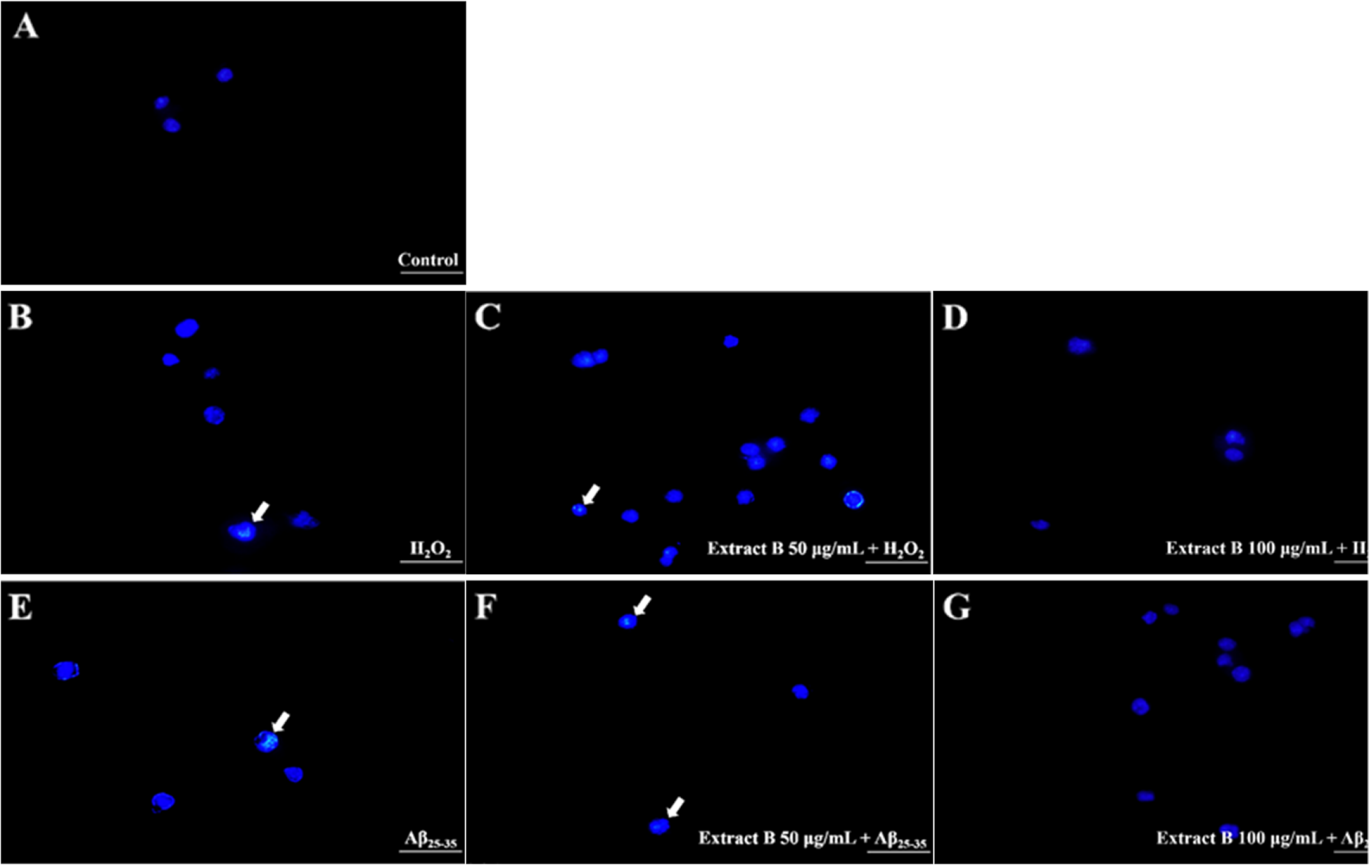
Protective effects of *G. lucidum*-fermented crop extract B against H_2_O_2_- or Aβ_25-35_-induced apoptosis in SH-SY5Y cells. **A** The representative image of non-treated SH-SY5Y cells stained with Hoechst 33,342 is shown. The experimental setup involved pretreating the cells with *G. lucidum* extract B at two different concentrations, namely 50 μM or 100 μM, for a duration of 6 h. Subsequently, the cells were exposed to either (**B**, **C**, **D**) H_2_O_2_ (150 μM) or (**E**, **F**, **G**) Aβ_25-35_ (10 μM) for an additional 24 h. Morphological apoptosis was determined by staining with Hoechst 33,342. Arrow heads indicate apoptosis cells, with condensed chromatin, causing an increase in fluorescence intensity. The scale bar represented 50 μm

In H_2_O_2_*-*treated SH-SY5Y cells, pre-treatment of non-fermented crop extract had no impact on improving cell viability (Fig. 2A). However, pre-treatment of extract A (10–25 μg/mL), extract B (100 μg/mL) and extract C (50–100 μg/mL) statistically increased cell viability compared to non-pretreatment (Fig. 2B-D). In Aβ_25-35_*-*treated cells, all of these fermented crop extracts (10–500 μg/mL) showed better effects on improving cell viability than that in H_2_O_2_*-*treated cells (Fig. 3). As extract B improved cell viability in a concentration-dependent manner (0–100 μg/mL), the preventive effect of extract B against H_2_O_2_- or Aβ_25-35_-induced apoptosis was further observed by Hoechst 33,342 staining. Compared to that in the control group, the condensed chromatin in apoptotic cells with bright fluorescence was observed in H_2_O_2_- or Aβ_25-35_-treated cells (Fig. 4A vs. 4B and 4E). However, less number of cells with bright fluorescence could be observed in cells pre-treated with extract B, especially at the concentration of 100 μg/mL (Fig. 4D and 4G). Therefore, this preliminary result may indicate that pretreatment with *Ganoderma* fermented crop extract may improve neuronal cell viability by preventing H_2_O_2_ or Aβ_25-35_-induced cell apoptosis. The anti-apoptotic activity and mechanism of fermented crop extracts in preventing apoptosis induced by H_2_O_2_ or Aβ_25-35_ merit further exploration in the future.

### Impact of *G. lucidum*-fermented crop extracts on the activity of intracellular antioxidant enzymes in H_2_O_2_- or Aβ_25-35_-treated SH-SY5Y cells

**Fig. 5:**
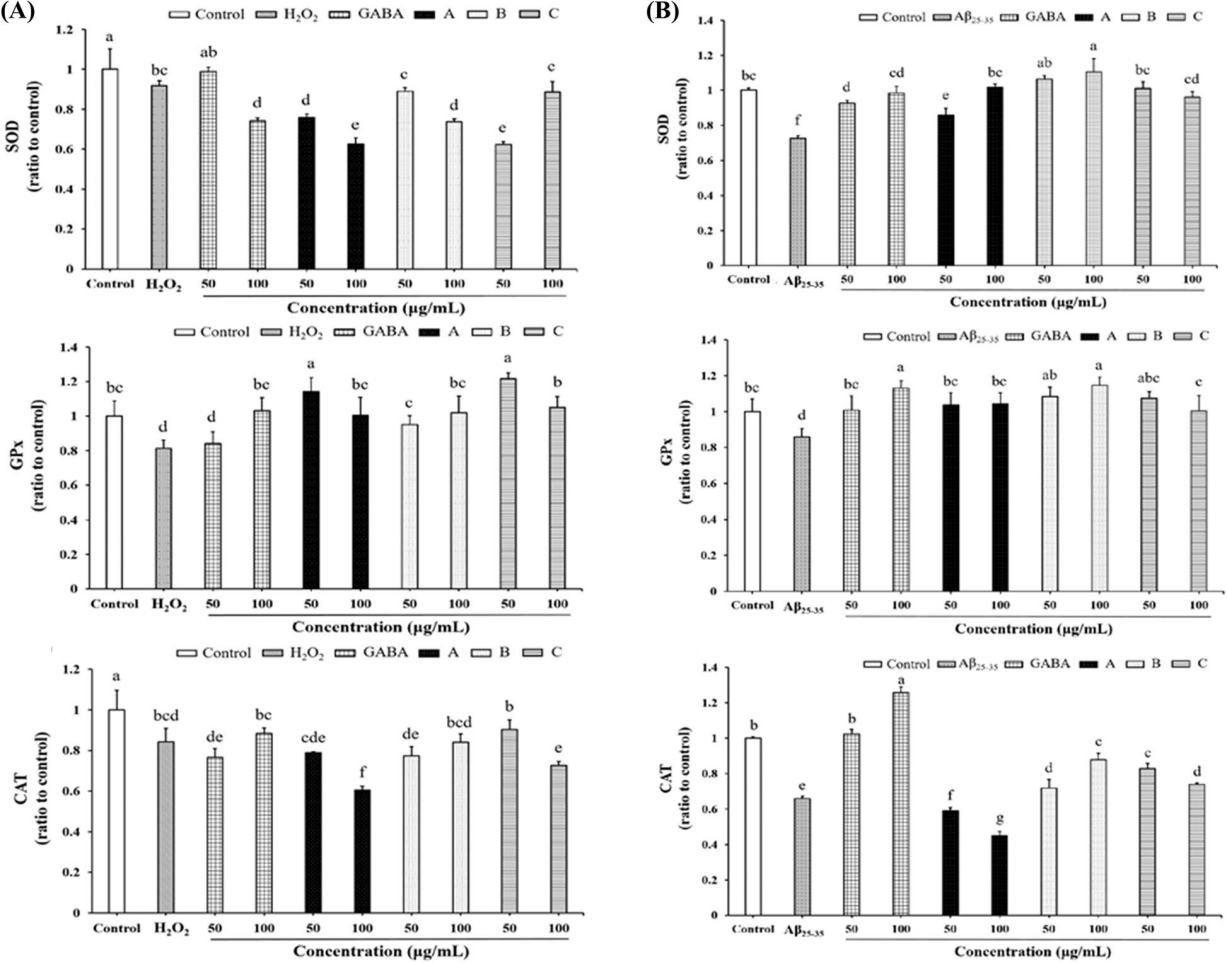
The impact of *G. lucidum*-fermented crop extracts on antioxidant enzyme depletion in SH-SY5Y cells was evaluated under two conditions: **(A)** H_2_O_2_- and **(B)** Aβ_25-35_-induced oxidative stress. The cells were pretreated with either GABA standard or various *G. lucidum* extracts for 6 h, followed by exposure to H_2_O_2_ (150 μM) or Aβ_25-35_ (10 μM) for 6 h (GPx and CAT) or 12 h (SOD) to measure the activity of antioxidant enzymes, as described in the Materials and methods section. The data were presented as mean ± SD from three independent experiments. Different letters (a-f) displayed within the same figure indicate significant differences (*p* < 0.05) among the various treatment groups

As the above results indicate a close association between the antioxidant and cytoprotective effects of *G. lucidum-*fermented crop extracts, the impact of extracts on intracellular antioxidant enzymes was further investigated in H_2_O_2_- or Aβ_25-35_-treated SH-SY5Y cells. First, time-course experiments were conducted to understand the time when SOD, GPx and CAT activities were altered in SH-SY5Y cells after H_2_O_2_ or Aβ_25-35_ treatment. A decrease in the activities of SOD, GPx and CAT beginning at 12, 12 and 6 h post-treatment of H_2_O_2_, respectively, was observed in SH-SY5Y cells (Fig. S4A). Activities of SOD, GPx and CAT were also obviously declined at 24 h post-treatment of Aβ_25-35_ (Fig. S4B). Following the above results, the impact of various extracts on the activities of intracellular antioxidant enzymes was determined at the anticipated time points. Neither GABA nor extracts treatment reversed H_2_O_2_-induced SOD and CAT decrement (Fig. 5A). However, the activity of GPx was up-regulated in cells pre-treated with extract A, extract B and extract C (Fig. 5A). In Aβ_25-35_-treated cells, all of these extracts could reversed the depletion of SOD and GPx activities (Fig. 5B). Although extract A had no influence on augmenting the activity of CAT, both extract B and extract C prevented against Aβ_25-35_–induced CAT decrement (Fig. 5B).

### Influence of *G. lucidum*-fermented crop extracts on the activity of acetylcholinesterase (AChE) in H_2_O_2_- or Aβ_25-35_-treated SH-SY5Y cells

**Fig. 6:**
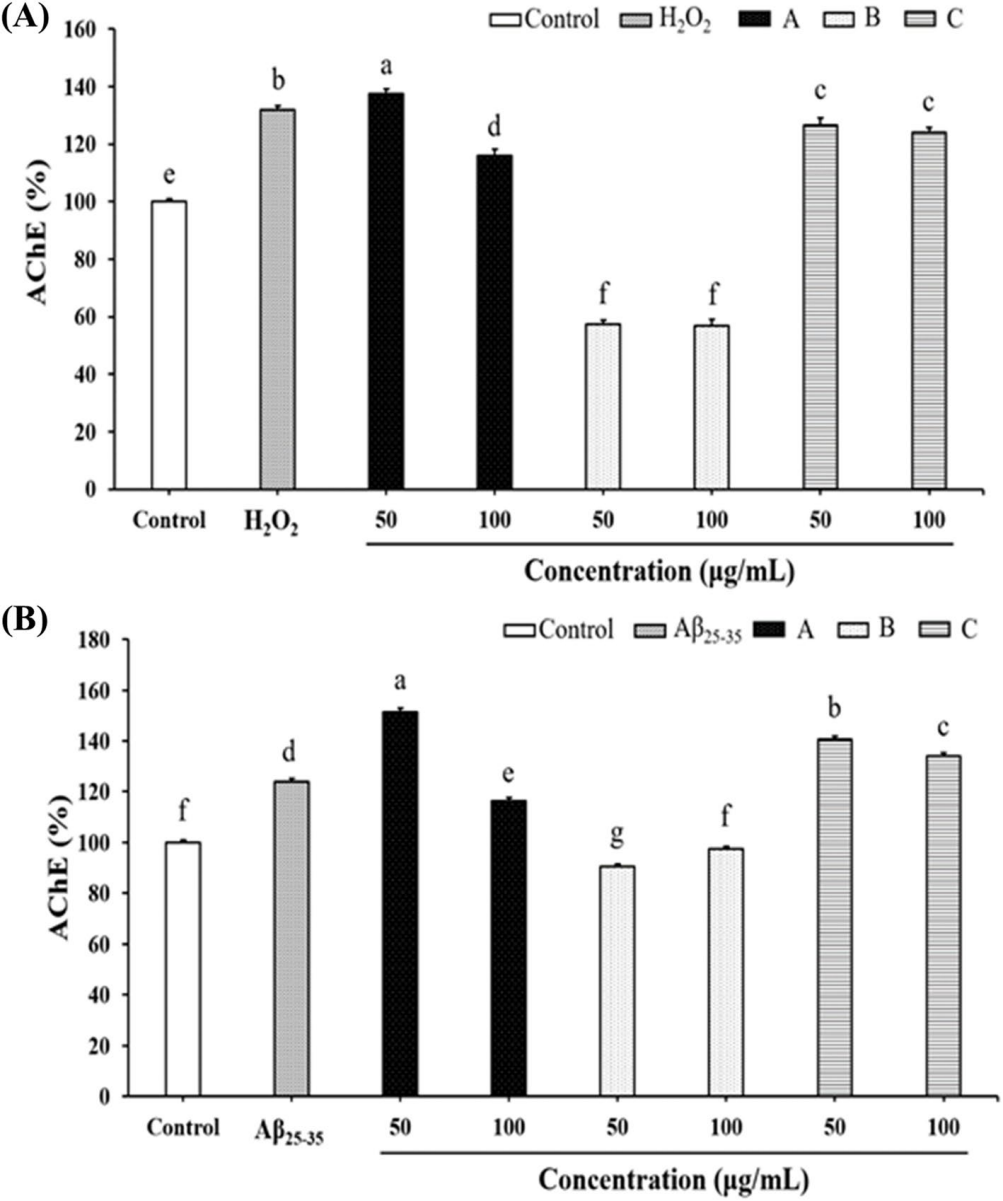
The study investigated the influence of *G. lucidum*-fermented crop extracts on acetylcholinesterase (AChE) activity in two conditions: (**A**) H_2_O_2_- and (**B**) Aβ_25-35_- treated SH-SY5Y cells. The cells were pre-treated with various *G. lucidum* extracts for 6 h, followed by exposure to H_2_O_2_ (150 μM) or Aβ_25-35_ (10 μM) for 24 h. AChE activity was determined as described in the Materials and methods section. The data were presented as mean ± SD from three independent experiments. Different letters (a-f) within the same group and figure indicate significant differences (*p* < 0.05) among the treatment groups

Since a common feature in the AD brain is the presence of AChE, which is commonly associated with β-amyloid plaques and oxidative stress, we finally investigated the influence of various extracts on the AChE activity in H_2_O_2_- or Aβ_25-35_-treated SH-SY5Y cells [28]. As shown in Fig. 6A and 6B, both H_2_O_2_ and Aβ_25-35_ treatment markedly enhanced the AChE activity. Although both extract A and extract C had limited impact on AChE activity, pre-treatment of extract B significantly reduced the AChE activity, indicating extract B as a potent AChE inhibitor (Fig. 6A & B).

## Discussion

Generally, it takes 3–5 months to artificially cultivate *G. lucidum* fruiting bodies on solid medium, and only 2–3 weeks to culture *G. lucidum* mycelium with shaking. [17]. In this study, we used an edible crop mixture as a solid medium to culture *G. lucidum* mycelium. Since it is only cultivated until the mycelium grows and not until the fruiting body grows, the time can be effectively shortened to 3 weeks. As demonstrated by Lin et al. [20], the presence of *G. lucidum* mycelia monitored by the amount of ergosterol contribute to increase the content of protein, lipid, ash (Table 1) and total glucan (Table 2) in fermented crop product. Additionally, *G. lucidium* mycelia in the solid medium can produce triterpenes and GABA (Table 2), which is rare in *G. lucidium* mycelia obtained by shaking culture. Furtherly, the data demonstrating protective effects of *G. lucidum*-fermented crop extracts on oxidative stress and Aβ_25-35_-induced neuronal damage indicate that fermentation of *G. lucidum* is an essential process to generate bioactive components contributing to neuroprotective activity. Most importantly, the content of biologically active ingredients contained in fermented crop extracts was addressed, which may help clear their free radical scavenging, antioxidant and anti-apoptotic properties, thus providing critical insights into the mechanisms of action behind their effects.

Previous studies have reported the potential of *G. lucidum* extracts on ameliorating AD and pointed out the major bioactive components. For example, Huang et al*.*[14] demonstrated that *G. lucidum* water extract could alleviate cognitive defects in transgenic AD mice and suggested that polysaccharides, but rather than protein and uronic acid, was the major bioactive ingredient contained in the water extract. In the study of Lai et al., the powder of *G. lucidum* aqueous extract contained 68.1% carbohydrate and 20.9% protein, and galactose and glucose were the major carbohydrates [15]. Treatment of rat primary cortical neurons with the aqueous extract significantly reduced Aβ-induced synaptic toxicity [15]. In fact, the extraction methods employed in these studies hardly isolate pure compounds. Therefore, the involvement of other residue ingredients in the biological activity of *G. lucidum* extracts could not be excluded.

So far, the association between neuroprotective ingredients of *G. lucidum* and their action mechanisms is not fully understood. As the potential of polysaccharides, triterpenes and GABA for the treatment of AD has been mentioned, we conducted different extraction methods to obtain triterpenes-, GABA- and polysaccharides-enriched extracts. In consistence with the study of Sun et al. showing neuroprotective effects of *G. lucidum* polysaccharides against oxidative stress-induced neuronal apoptosis, treatment of extract C, containing the highest level of total glucan among the extracts, reduced H_2_O_2_- or Aβ_25-35_-induced ROS production, lipid peroxidation and even improved cell viability in SH-SY5Y cells [12]. The increased levels of intracellular SOD, GPx and CAT are suggested as one of the potential mechanisms.

Beside polysaccharides, triterpenes, due to their cholinesterase inhibitory activities, are also considered one of the main components of *G. lucidum* to possess beneficial effects against AD [41]. Kaur et al. have indicated that the action mechanism of *Ganoderma*–mediated anti-amnesic effects is involved to their anti-acetylcholinesterase and antioxidant effects [42]. In addition, it has been explored that treatment of *Ganoderma* triterpenes could alleviate the cognitive impairment of AD mice by inhibiting apoptosis, reducing oxidative damage, promoting SOD expression and inhibiting neuronal MDA production [16]. Concordantly, our results showed that treatment of extract B, containing the highest level of triterpenes among these extracts, exhibited most potent effects on attenuating H_2_O_2_- or Aβ_25-35_-induced cell apoptosis, reversing the diminished activity of SOD, suppressing MDA production and inhibiting AChE activity. The cholinergic hypothesis is the earliest theory regarding the pathogenesis of AD, and previous studies have indicated the cholinergic mechanism of *Ganoderma* for its anti-amnesic effect [42]. Therefore, we investigated the cholinergic mechanism in this study. However, further investigations will be necessary to understand whether the neuroprotective effect of *G. lucidum*-fermented crop extract involves other mechanisms, including synergistic effects.

Significant reductions in GABA levels have been described in severe cases of AD, and several GABAergic drugs have been tested for efficacy in attenuating or reversing various features and symptoms of AD [18, 43]. In parallel with previous studies showing the antioxidant and anti-apoptotic potentials of GABA, the results of current study reveal that both GABA and extract A, containing the highest level of GABA among these extracts, obviously improved cell viability and up-regulated the activity of intracellular antioxidant enzymes in H_2_O_2_- or Aβ_25-35_-treated SH-SY5Y cells [44, 45]. Interestingly, treatment of extract C, containing limited level of GABA, also improved cell viability. Accordingly, it is suggested that the antioxidant and anti-apoptotic activity of fermented crop extracts was not only owing to GABA but also resulted from other bioactive ingredients.

Both phenolics and flavonoids are well-known antioxidants with the capacity of increasing intracellular SOD, GPx and CAT activities [46, 47]. Although extract A contained the highest levels of phenolics and flavonoids among these extracts, the efficacy of extract A in decreasing ROS production and increasing antioxidant enzyme activity was not better than that of the other extracts. It is suggested that the antioxidant and anti-apoptotic activity of *G. lucidum*-fermented crop extracts not only resulted from one or the major bioactive ingredient, but the interaction between these bioactive ingredients may play a crucial role in their neuroprotective properties. This issue warrants a more comprehensive investigation to elucidate the pharmacodynamic and even pharmacokinetic interaction of these bioactive ingredients, which will also help us to understand why all of the fermented crop extract-mediated effects were not in a concentration-dependent manner. On the other hand, oxidative stress occurs when the balance between antioxidants and ROS are disrupted due to either depletion of antioxidants or accumulation of ROS [48]. Therefore, it is reasonable to observe that the treated cells with the highest activity of antioxidant enzyme were far from that showed the lowest ROS and MDA production and even the best cell viability detected at the anticipated time points.

## Conclusion

Our findings provide the first evidence to demonstrate the neuroprotective effects of solid-state *G. lucidum*-fermented crop extracts against H_2_O_2_- or Aβ_25-35_-induced oxidative damage in neuronal cells. Free radical scavenging capacity, antioxidant activity and AChE inhibitory effects are involved in the action mechanism of neuroprotection. Glucan, triterpenes, GABA, phenolics and flavonoids are suggested as the major bioactive components contributing to neuroprotective effects. Although further efforts are required to verify the in vivo activity of *G. lucidum*-fermented crop extracts and the interaction between the bioactive ingredients, these results provide new clues for future therapeutic research of neurodegenerative diseases, especially AD.

### Supplementary Information

**Supplementary Material 1.**

## Data Availability

Upon request, the data are available from the corresponding author.

## Abbreviations

ABTS: 2,2'-Azino-bis(3-ethylbenzothiazoline-6-sulfonic acid)
AChE: Acetylcholinesterase
AD: Alzheimer's disease
CAT: Catalase
DCFH-DA: Diacetyldichlorofluorescein
DPPH: 1,1-Diphenyl-2-picrylhydrazyl
GABA: γ-Aminobutyric acid
GPx: Glutathione peroxidase
IC_50_: Half maximal inhibitory concentration
MDA: Malondialdehyde
MTT: 3-(4,5-Dimethylthiazol-2-yl)-2,5-diphenyl-tetrazolium bromide
O.D.: Optical density
PBS: Phosphate-buffered saline
QE: Quercetin equivalents
ROS: Reactive oxygen species
SOD: Superoxide dismutase
TBARS: thiobarbituric acid reactive substance
SD: Standard deviation
