# Supplementary material for: Protective effect of Ganoderma lucidum-fermented crop extracts against hydrogen peroxide- or β-amyloid-induced damage in human neuronal SH-SY5Y cells
Source: BMC Complement Med Ther. 2024 Apr 5;24:148. doi: 10.1186/s12906-024-04409-1 (PMC10996153; doi:10.1186/s12906-024-04409-1)
Supplement: Supplementary file 1 — Supplementary Material 1. [file 12906_2024_4409_MOESM1_ESM.docx]

**Protective effect of *Ganoderma lucidum*-fermented crop extracts against hydrogen peroxide- and β-amyloid-induced damage in human neuronal SH-SY5Y cells**

Chung-Hsiung Huang^1^, Yu-Ting Liao^1^, Chien-Li Chen^1^ and Guo-Jane Tsai^1,2*^

*^1^Department of Food Science, National Taiwan Ocean University, Keelung, Taiwan*

*^2^Center for Marine Bioscience and Biotechnology, National Taiwan Ocean University, Keelung, Taiwan*

^*^Corresponding author: Dr. Guo-Jane Tsai

Department of Food Science, National Taiwan Ocean University, 2 Pei-Ning Road, Keelung, 202, Taiwan, ROC

Tel: +886-2-2462-2192#5150

Fax: +886-2-2462-7954

E-mail: [b0090@mail.ntou.edu.tw](mailto:b0090@mail.ntou.edu.tw)


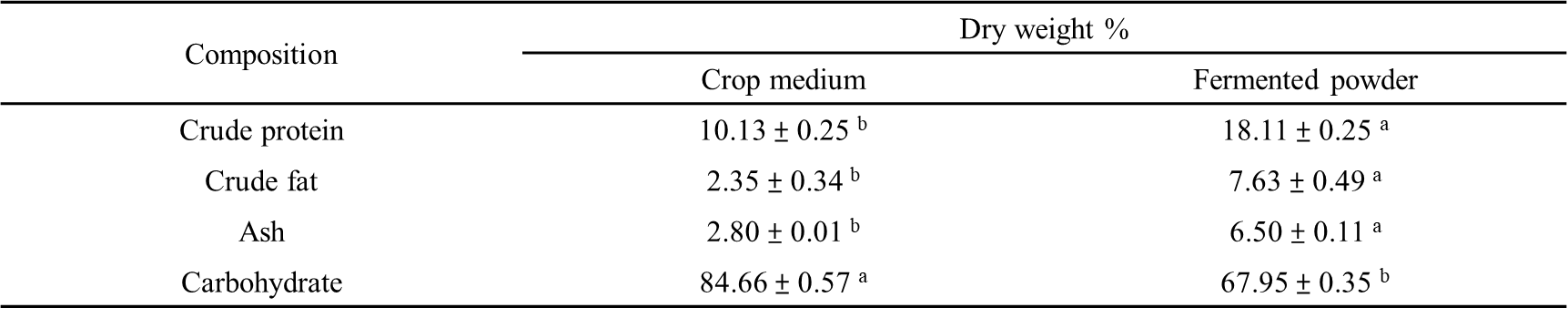
Table S1. Composition (dry weight basis) of crop medium and *G. lucidum*-fermented crop powder.

Data were expressed as mean ± SD from triplicate experiments. Different letter ^a-b^ in the same group mean significant difference (*p*<0.05). Carbohydrate was equal to 100% minus crude protein, crude fat and ash.

**Fig. S1** HPLC profiles of GABA content in **(A)** GABA standard, **(B)** crop medium, **(C)** *G. lucidum*-fermented crop powder, **(D)** *G. lucidum*-fermented crop extract A, **(E)** extract B and **(F)** extract C.

**Fig. S2** Cell viability of SH-SY5Y cells after treatment with **(A)** crop medium extract, **(B)** *G. lucidum*-fermented crop extract A, **(C)** extract B and **(D)** extract C (0-500 μg/mL) for 24 h determined by MTT assay. Data are expressed as mean ± SD from triplicate experiments. ^*^Significant difference versus non-treatment (*p*< 0.05).

**Fig. S3** Cell viability of SH-SY5Y cells after Aβ_25-35_ or H_2_O_2_ treatment. Fluorescent microscope images of Aβ_25-35_ plaques after **(A)** 0 day and **(B)** 7 days cultivation by staining with thioflavin T, and scale bar represented 100 μm. Cell viability of SH-SY5Y cells after treatment with either **(C)** H_2_O_2_ (0-300 μM) or **(D)** Aβ_25-35_ (0-15 μM) for 24 h determined by MTT assay. Data are expressed as mean ± SD from triplicate experiments. ^*^Significant difference versus non-treatment (p< 0.05).

**Fig. S4** Time course analysis of superoxide dismutase (SOD), glutathione peroxidase (GPx) and catalase (CAT) activity in SH-SY5Y cells treated with **(A)** H_2_O_2_ (150 μM) or **(B)** Aβ_25-35_ (10 μM). Data are expressed as mean ± SD from triplicate experiments. ^*^Significant difference versus 0 h of treatment time (p< 0.05).
